# Evaluation of the Performance of Different Types of Fibrous Concretes Produced by Using Wollastonite

**DOI:** 10.3390/ma15196904

**Published:** 2022-10-05

**Authors:** Maciej Dutkiewicz, Hasan Erhan Yücel, Fatih Yıldızhan

**Affiliations:** 1 Faculty of Civil and Environmental Engineering and Architecture, Bydgoszcz University of Science and Technology, 85-796 Bydgoszcz, Poland; 2 Civil Engineering Department, Engineering Faculty, Niğde Ömer Halisdemir University, Niğde 51240, Turkey; 3 Civil Engineering Department, Engineering Faculty, Gaziantep University, Gaziantep 27310, Turkey

**Keywords:** wollastonite, fibrous concrete, mechanical properties, durability properties

## Abstract

Production of cement and aggregate used in cement-based composites causes many environmental and energy problems. Decreasing the usage of cement and aggregate is a crucial and currently relevant challenge to provide sustainability. Inert materials can also be used instead of cement and aggregates, similar to pozzolanic materials, and they have positive effects on cement-based composites. One of the inert materials used in cement-based composites is wollastonite (calcium metasilicate-CaSiO_3_), which has been investigated and attracted attention of many researchers. This article presents state-of-the-art research regarding fibrous concretes produced with wollastonite, such as mortars, conventional concrete, engineered cementitious composites, geopolymer concrete, self-compacting concrete, ultra-high-performance concrete and pavement concrete. The use of synthetic wollastonite, which is a novel issue, its high aspect ratio and allowing the use of waste material are also evaluated. Studies in the literature show that the use of wollastonite in different types of concrete improves performance properties, such as mechanical/durability properties, and provides environmental–economic efficiency. It has been proven by studies that wollastonite is a material with an inert structure, and, therefore, its behavior is similar to that of a fiber in cementitious composites due to its acicular particle structure.

## 1. Introduction

Concrete, which is the most used construction material thanks to its superior properties [1], increases production every year, and it is estimated that it reaches approximately 11 billion metric tons annually [2,3,4]. Cement, which constitutes 10–15% of concrete by volume [4,5], is the most expensive component of concrete and causes the main environmental and energy consumption problems [6,7]. For example, cement, which is responsible for over 5% of global CO_2_ emissions [8,9], generates approximately 50% of these emissions during the calcination phase of carbonate raw material [9,10,11]. Because global CO_2_ emissions reached approximately 30 billion tons with an increase of 2% in 2020 [12], along with the increasing global warming effect, production of cement must be reduced. In addition to this problem, environmental and energy problems in the supply and transportation of aggregate must be considered for concrete production. The usage of pozzolanic additives, such as fly ash, blast furnace slag and silica fume, which have binding properties, has been experimented with in many studies to reduce cement or aggregate production. These pozzolanic additives have enhanced the performance properties of concrete and have reduced devastating environmental problems in cement-aggregate production. Not only pozzolanic materials but also inert materials can be used as alternatives to cement or aggregates [13].

**Figure 1:**
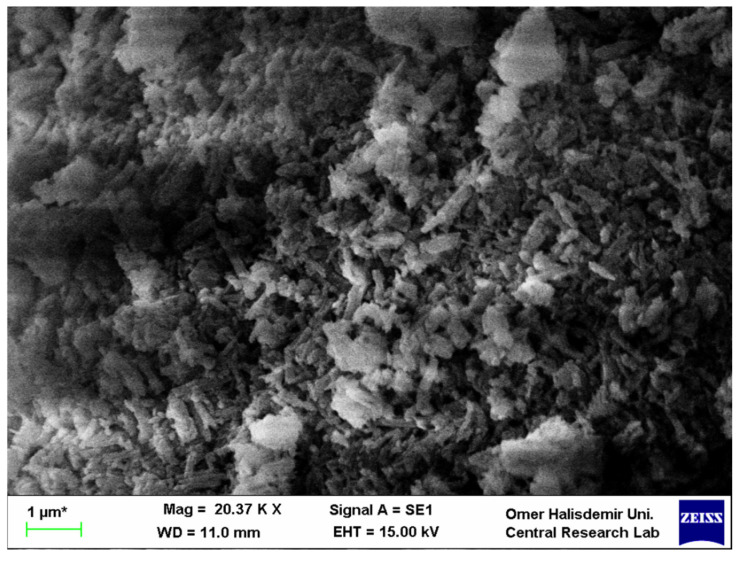
SEM image of wollastonite.

One of the most used inert materials is wollastonite. Wollastonite was discovered in 1886 by British chemist Sir Wollaston and was named after him [14,15]. Wollastonite, which consists of two main basic components, 48.3% CaO and 51.7% SiO_2_, has a molecular weight of 116.2 [15,16,17]. The chemical composition of wollastonite is provided in Table 1. Small amounts of Al_2_O_3_, Fe_2_O_3_, MgO and Na_2_O could also be available in the chemical composition of wollastonite. Wollastonite is also called calcium metasilicate, and the chemical formula of this mineral is CaSiO_3_ [18]. A scanning electron microscopy (SEM) image of wollastonite is shown in Figure 1. It is a white-colored natural mineral that occurs as a result of a reaction between limestone and silica at 400 °C–450 °C, and it has an acicular or needle-like shape [13,14,19,20,21]. The melting point of wollastonite is 1540 °C [15], the modulus of elasticity of wollastonite is approximately in the range of 300 to 530 GPa and the tensile stress is approximately in the range of 2700 to 4100 Mpa [13]. Wollastonite has thermal stability, low dielectric constant, low dielectric loss, corrosion resistance and chemical inertness [22,23,24,25]. Therefore, wollastonite has a very wide usage area, such as ceramic, dental, paint plastic and many other fields [26,27,28]. Moreover, wollastonite can enhance environmental sustainability and durability properties in cement-based composites [13], and it has the capability to decrease the cost of cement-based composites. However, its availability is limited because it is a natural mineral [14]; thus, researchers have focused on production of synthetic wollastonite [7,13,29].

Wollastonite can be artificially synthesized with different materials. For example, it was produced with eggshell and silica [30], rice husk ash, silica ferrochrome, diatomite, quartz and calcined marble tailings in the literature [14,31]. Protection of natural wollastonite resources and use of waste material provide a significant advantage for synthetic wollastonite. There are three different methods for production of synthetic wollastonite. These methods are the wet method, which has lower than 200 °C and high pressure; the solid-state reaction method, which involves reaction of silica with calcium oxide or calcium carbonate at a temperature higher than 800 °C and liquid phase reaction method, which is higher than 1400 °C [13]. Each method contains different advantages and disadvantages in itself. However, the aspect ratio of synthetic wollastonite with acicular particle structure is not as high as that of natural wollastonite. Therefore, a new technique that brings together the three-step process, which includes the mechanochemical process, hydrothermal process and solid-state reaction, was developed [13]. Wollastonite is available in nature with aspect ratios of 3:1 to 20:1 [32], but wollastonite with an aspect ratio of 23:1 [13] and even 44:1 [33] was developed in productions synthetically. Therefore, the use of synthetic wollastonites in cement-based composites or in different fields is precious and has a promising future to study.

In recent years, many studies have been conducted on the use of both natural wollastonite and synthetic wollastonite in cement-based composites. Especially, the use of synthetic wollastonite is novel and contains valuable findings for future studies regarding cement-based composites because of the high aspect ratios of acicular particle structure. In this study, use of wollastonite in mortar, conventional concrete, engineered cementitious composites, geopolymer concrete, self-compacting concrete, ultra-high-performance concrete and pavement concrete was investigated. The effects of wollastonite usage in different cementitious composites on the physical, mechanical and durability properties were presented and discussed. In addition, general evaluations were conducted according to these discussions. Finally, suggestions were put forward regarding possible future research directions.

## 2. Cement Paste and Mortar

**Figure 2:**
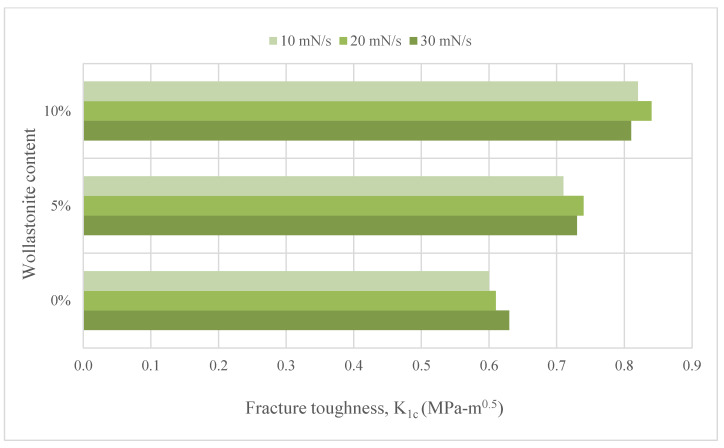
Fracture toughness of wollastonite content based on Ref. [34].

**Figure 3:**
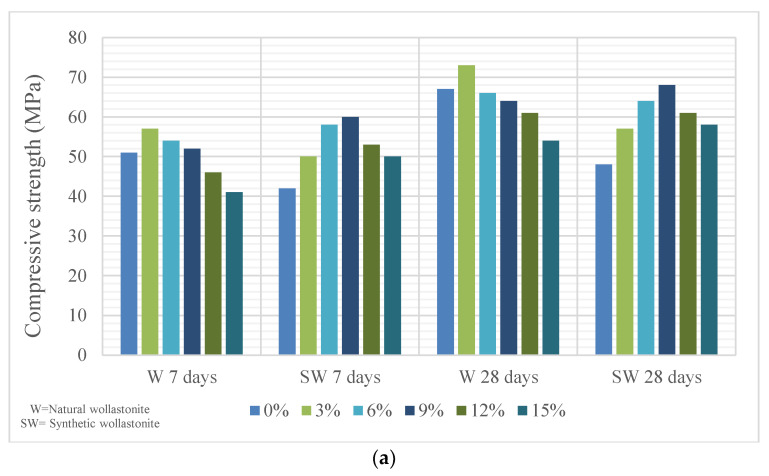

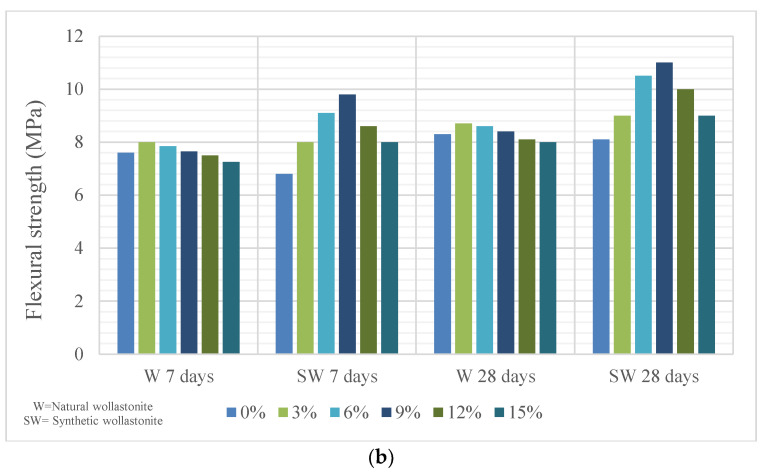
(**a**) Compressive and (**b**) flexural strengths of natural wollastonite and synthetic wollastonite based on Refs. [13,35].

Cement paste consists of cement and water; mortar consists of cement, water and fine aggregate. Studies on cement paste or mortar were performed in the form of using wollastonite replacement of sand and/or cement. Doner et al. [34] used 5% and 10% wollastonite by mass replacement of cement in their study. Figure 2 shows the fracture toughness of wollastonite content. They observed that the use of 5% and 10% wollastonite increased the fracture toughness by 17.88% and 33.71%, respectively. The toughening effects of wollastonite usage result from the acicular nature of wollastonite that significantly bridge cracks at the micro-level. In this way, it delays microcrack coalescence. In a study where natural wollastonite from 0% to 15% by 3% increments was used instead of cement [35], the best mixture containing 3% wollastonite performed better than the control mixture for capillary water permeability and gas permeability. The compressive and flexural strengths of the mixtures are provided in Figure 3. The strength increase in the use of 3% wollastonite could be explained by the acicular particle structure and high modulus of elasticity of the wollastonite. The use of wollastonite at rates of 6% or more could cause deterioration of the microstructure and decrease in strength.

**Figure 4:**
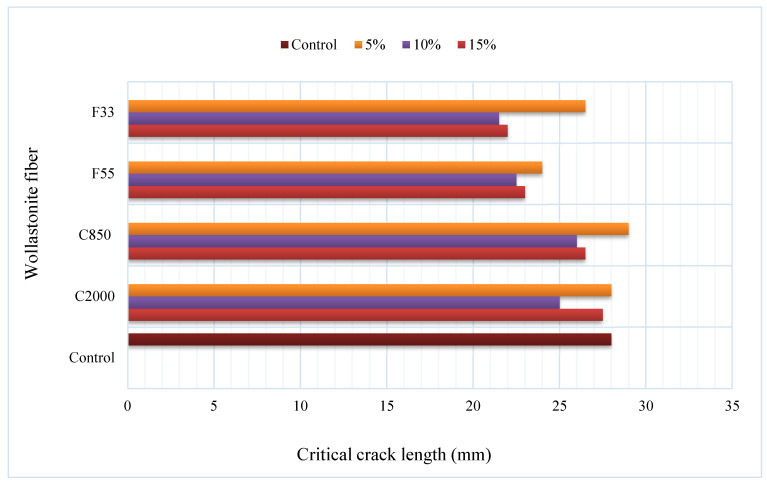
Critical crack length in cured 28 days based on Ref. [28].

**Figure 5:**
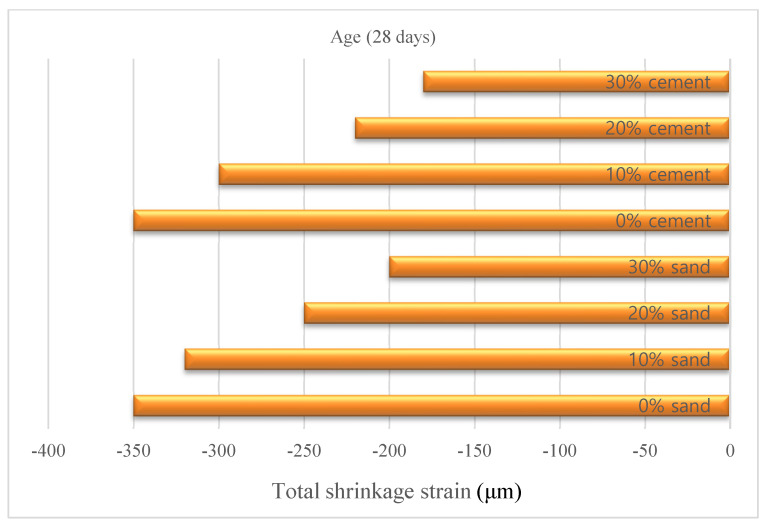
Total shrinkage strain based on Ref. [37].

In some studies, comparisons were performed by using wollastonite and an additional material. In a study in which the effect of wollastonite average particle size was taken into account, 3.5 µm and 9.0 µm average particle size wollastonite was used at a rate of 10%, 20%, 30%, 40% and 50% by mass in replacement of cement [36]. In addition, the results of wollastonite and limestone were compared by using limestone in the same proportions. The use of wollastonite decreased the workability of mortar mixes but increased cement hydration. Using 10% limestone reduced the compressive strength of the mortar by approximately 20%. For the same strength range, wollastonite-3.5 µm can be used in replacement of 40% cement. The use of wollastonite-3.5 µm instead of 30% of the cement reduced the 28-day compressive strength by only a rate of 10%. In other words, wollastonite can be used to replace high ratio of cement up to 30% in mixes without remarkably reducing strength. Dey et al. [28] took into account aspect ratio in addition to average particle size. They used 5% silica fume instead of cement to form a control mixture and also 5%, 10% and 15% wollastonite instead of cement; four grades of wollastonite fibers with average particle size ranging from 33 to 2000 µm, with aspect ratios varying from 3:1 to 20:1, were used. The results of the study showed that the wollastonite fibers moderately increased the compressive strength and also significantly increased the fracture strength and toughness and, finally, enhanced ductility. At optimum dosage, an increase in 28-day compressive strength up to 30%, an increase in flexural strength of up to 41% and an increase in toughness of up to 147% were observed compared to the control mixture. The crack results of this study are provided in Figure 4. C refers to coarse grades, F refers to fine grades and the number refers to the average particle size in Figure 4. Wollastonite increased crack growth resistance. This can be attributed to matrix packing and its bridging of microcracks that lead to delayed microcrack coalescence thanks to usage of wollastonite. Moreover, the optimum replacement was dependent on the wollastonite fiber type. In a study where wollastonite and additional material were evaluated instead of cement, Ransinchung and Kumar [20] investigated the effect of wollastonite, microsilica and combination of wollastonite + microsilica on cement-based composites. Wollastonite increased the initial–final setting time, and 10% wollastonite+7.5% microsilica combination provided the highest strength value. The combination of 15% wollastonite+7.5% microsilica also showed higher strength than the control mix. In a study in which wollastonite was used instead of both sand and cement, 10%, 20% and 30% wollastonite were used instead of sand and cement, thus forming seven mixtures including the control mixture [37]. The initial setting time increased by using wollastonite, but this increase had a negligible effect on cement replacement. The use of wollastonite up to 20% instead of sand increased the compressive and flexural strength. When using wollastonite instead of 20% sand, 28-day compressive strength increased by 45%, and flexural strength increased by 28% compared to the control mixture. The mechanical strength of using wollastonite instead of 30% sand was also higher than the control mixture. When using wollastonite instead of 10% cement, the compressive strength decreased by 12% and the flexural strength decreased by 2% compared to the control mixture. The strengths were much lower when wollastonite was used instead of 20–30% cement. The total shrinkage strain is displayed in Figure 5. Usage of wollastonite decreased the drying shrinkage with 30% cement replacement and 30% sand replacement at the rates of 47% and 44%, respectively. From the studies, the increase in strength and durability with the use of wollastonite was attributed to the effect of densifying the microstructure of the matrix and the filling effect of wollastonite. Leeman et al. [38] used carbonated wollastonite clinker instead of 30% of cement by weight. They found that this mixture increased the Si/Ca ratio by 15% compared to the control mixture but decreased the 28-day compressive strength by 7.8%.

**Figure 6:**
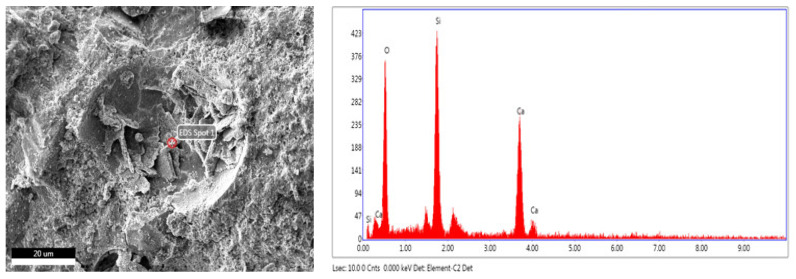
SEM images and EDX analysis of synthetic wollastonite.

**Figure 7:**
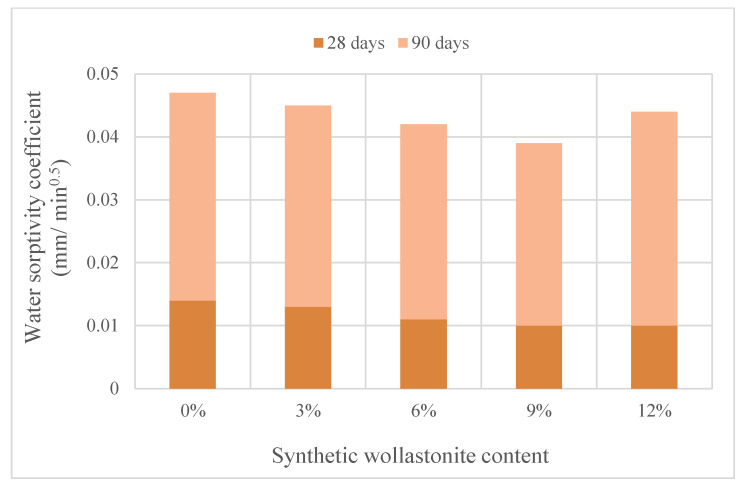
The water sorptivity coefficient and synthetic wollastonite content based on Ref. [7].

In addition, studies on the use of synthetic wollastonite on mortars were also carried out. Kalkan et al. [29] used 0%, 0.5%, 1%, 2%, 5%, 10%, 15% and 20% synthetic wollastonite instead of cement. The use of wollastonite reduced the workability, and the initial and final setting times were delayed. Water absorption decreased up to 2% replacement. It enhanced at 2% replacement in the mechanical properties of mortars. Moreover, 5% replacement can be a usable ratio and indicated higher strength than 0% replacement. The increase in flexural strength with the use of wollastonite was higher than the percent compressive strength. Moreover, thermal conductivity decreased as replacement ratio increased. Yücel and Özcan [13] formed a total of six mixtures, including a control mixture and usage of wollastonite from 0% to 15% by 3% steps of increment replacing cement. Slump flow diameter decreased with use of wollastonite. Figure 3 shows the compressive and flexural strengths of synthetic wollastonite. The compressive and flexural strengths increased up to 9% usage and reached their maximum value at 9%. In addition, the mechanical properties of the 12% and 15% replacement mixtures provided better results than the control mixture. Synthetic wollastonite fills the porous structure of cement and forms a more compact mortar [13,37,39]. When natural wollastonite and synthetic wollastonite are compared, according to Figure 3, it is observed that the optimum dosage and rate of increase are higher for synthetic wollastonite. The SEM images and energy dispersive X-ray (EDX) analysis of synthetic wollastonite are presented in Figure 6. The acicular structure of synthetic wollastonite is also shown in Figure 6. The enhancement in the mechanical properties can be explained by the physical property (acicular structure) of synthetic wollastonite acting as a fiber in the matrix. Moreover, these results supported that synthetic wollastonite is an inert material and, thus, does not show any chemical reaction. CaCO_3_ tended to decrease with the addition of synthetic wollastonite. Öz and Güneş [7], on the other hand, examined the effects of synthetic wollastonite on high-performance mortars. A total of five mixtures were formed: control mixture and usage of synthetic wollastonite at 3%, 6%, 9% and 12% ratios instead of cement. Use of wollastonite reduced workability. The compressive and flexural strengths increased up to 9% usage. At 12% usage, the mechanical properties were worse than those of the control mix. The water sorptivity coefficient is provided in Figure 7. The performance properties of the water sorptivity coefficient, rapid chloride permeability and gas permeability, which were tested for durability, improved up to 9% usage. Thanks to the synthetic wollastonite filling effect, the microstructure in the cement matrix condensed. Therefore, synthetic wollastonite reduced the water absorption of the material and provided pore discontinuity in the cement [7,20,32,39]. While the Yücel and Özcan [13] 12% synthetic wollastonite mixture was better than the control mixture, it was worse than the control mixture in the study by Öz and Güneş [7]; this difference is due to the w/c ratio. Usage of synthetic wollastonite up to a 10% ratio also indicates very valuable findings for future studies. When the usage of wollastonite on cement paste and mortar is examined, it is found that wollastonite was used instead of sand and/or cement; further, these uses were tested with wollastonite + different material combinations. In addition, it is observed that synthetically produced wollastonites were also used. Cement paste or mortars that have higher mechanical and durability properties were formed with usage of natural or synthetic wollastonite.

## 3. Conventional Concrete

**Figure 8:**
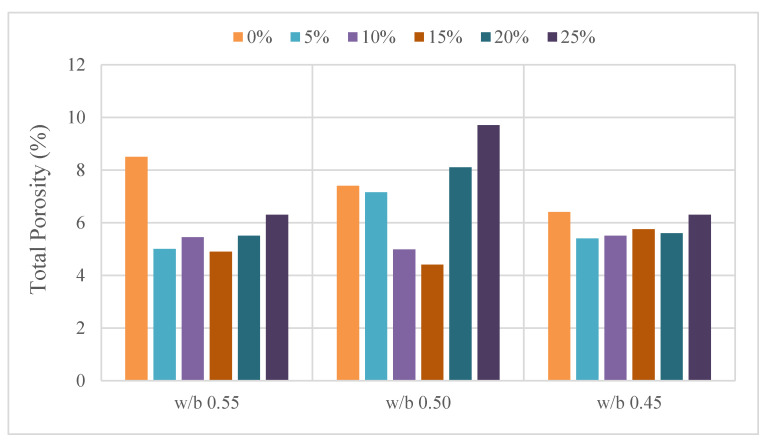
Porosity and wollastonite replacement based on Ref. [32].

Concrete is formed by mixing cement, water, coarse aggregate, fine aggregate, chemical additives and mineral additives at a certain rate. The usage of wollastonite in concrete was performed in three different ways: replacement of sand, cement and their combination. Kh [40] used 10%, 15% and 20% wollastonite by mass replacement of sand. A compressive toughness test, which is significant for quantitative analysis of the energy distributing ability of materials, was implemented for these mixtures. It was observed that the use of 15% wollastonite increased 23–25% in 28-day results compared to the control mixture. This increase was observed as 20% at 10% usage of wollastonite. In a study evaluating the durability properties, Aziza and Kh [41] used wollastonite replacement of 30% sand and found that it improved the durability properties by 32%. In a study where wollastonite was used in replacement of cement, 5% and 10% wollastonite were used [42]. It was determined that the use of 10% wollastonite increased the compressive toughness by 40% and the flexure toughness by 32%. These values were increased by 21% and 20% at 5% usage, respectively. In a study where the w/b ratio was taken into account in addition to the use of wollastonite replacement of cement, the compressive strength increased up to use of 10% wollastonite in all w/b ratios [32]. Flexural strength increased up to use of 15% wollastonite at 0.55 w/b ratio and increased up to use of 10% wollastonite at 0.50 and 0.45 w/b ratios. Porosity and wollastonite replacement is provided in Figure 8. As noted in other studies [7,13,20,32,39], the addition of wollastonite resulted in reduced pores and densification of the concrete microstructure. It was found that 10–15% wollastonite substitution instead of cement improves the durability properties of concrete, such as water permeability, porosity, carbonation, chloride diffusion and corrosion, in different w/b ratios.

In some studies for concrete, different material combinations were attempted, including wollastonite replacement of cement or replacement of cement+fine aggregate. Kalla et al. [43] examined the effect of the combination of wollastonite and fly ash replacement of cement, taking into account the w/b ratio. They used 40% fly ash (fixed ratio) instead of cement, as well as wollastonite from 0% to 25% by 5% steps of increment in replacement of cement. The mechanical properties increased up to use of 55% combination wollastonite (15%) + fly ash (40%) at a 0.55 w/b ratio and increased up to use of 60% combination wollastonite (20%) + fly ash (40%) at 0.50 and 0.45 w/b ratios. Additionally, permeability 55–60%, carbonation resistance 45%, diffusion 45–60%, corrosion 45–55% and shrinkage resistance at the rate of 55–60% wollastonite+fly ash combination enhanced its durability properties. It was found that the usage of wollastonite + fly ash combination in the range of 40–55% and wollastonite in the range of 5–15% affect the mechanical and durability properties of concrete positively. The wollastonite + fly ash combination was investigated in replacement of both cement and sand in another study [39]. It was stated that the mixture of fly ash instead of 20% cement + wollastonite instead of 10% sand increased the 28-day compressive strength by 28% and flexural strength by 36% compared to the mixture using only fly ash in replacement of cement at 20%. In addition, it was stated that the combination of fly ash in replacement of 20% cement + wollastonite in replacement of 10% sand and only wollastonite in replacement of 10% sand showed higher mechanical strength compared to the control mixture (no fly ash and no wollastonite). Finally, it was found that the usage of wollastonite (single or in combination with fly ash) reduces water absorption, drying shrinkage and abrasion loss. These findings obtained from the studies [39,43] show that fly ash and wollastonite could be a preferable alternative material combination. In another study, the use of 10% wollastonite in replacement of cement was taken as a fixed ratio, and, furthermore, waste granite fine was used at a rate of 10%, 20%, 30%, 40% and 50% in replacement of fine aggregate [44]. A mix design of concretes is provided in Table 2. Indeed, the 20% waste granite fine + 10% wollastonite combination mixture, which provided the best results, increased the compressive strength by 5.7% compared to the control mixture, and the 10% waste granite fine + 10% wollastonite combination and 30% waste granite fine + 10% wollastonite combination mixtures showed superior mechanical properties compared to the control mixture. Further, the 10–20% and 30% waste granite fine + 10% wollastonite combination mixture showed superior durability properties compared to the control mixture, but other mixtures (40% and 50% waste granite fine+10% wollastonite combination) showed worse properties than the control mixture. According to the findings obtained from the studies [32,43,44], generally, 10% was found as the optimum ratio for the usage of wollastonite in replacement of cement. Moreover, the use of 10–15% wollastonite has positive effects on durability properties [32,39,44]. When usage of wollastonite on concrete is examined, it is observed that wollastonite was used instead of sand and/or cement; further, these uses were tested with wollastonite + different material combinations, similar to mortar. Moreover, it is observed that waste materials were used in concrete. The use of a combination of wollastonite and waste material is crucial for forming sustainable concrete due to the reduction in cement use and recycling of waste material.

## 4. Engineered Cementitious Composite (ECC)

**Figure 9:**
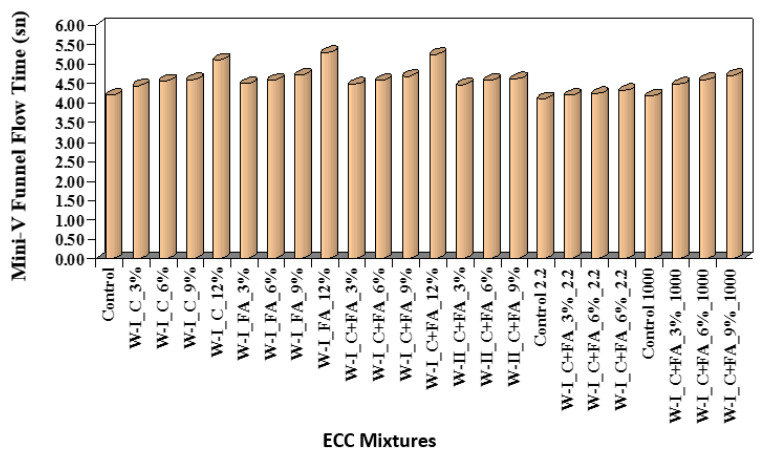
Mini-v-funnel flow time values of ECCs based on Ref. [33].

**Figure 10:**
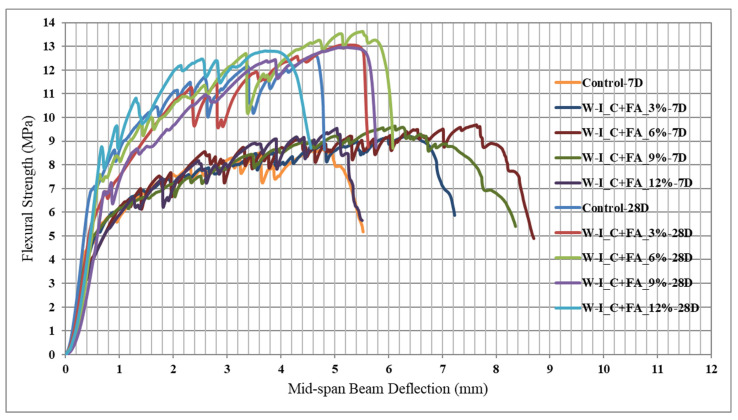
Typical flexural strength and mid-span beam deflection curve based on Ref. [33].

Engineered cementitious composite (ECC) is defined as a special type of high-performance fiber concrete and shows strain hardening similar to a ductile material after the first crack and has approximately 300 to 500 times greater tensile strain capacity compared to conventional concretes. In a study conducted for ECC, synthetic wollastonites with different high aspect ratios were used instead of cement, fly ash and cement + fly ash [33]. The mini-v-funnel flow time values of ECCs are provided in Figure 9. Wollastonite with a 44:1 aspect ratio which was used as a cement replacement decreased workability. The decrease in the workability of the ECC is mainly caused by the interlocking of the acicular particle structure of wollastonite. The usage of synthetic wollastonite instead of fly ash or cement + fly ash enhanced the mechanical performance up to 6% in terms of compressive strength and flexural performance. Typical flexural strength and mid-span beam deflection curves are shown in Figure 10. Using synthetic wollastonite with a 44:1 aspect ratio exhibited behavior similar to a fiber compared to natural wollastonite. Therefore, a remarkable enhancement in ductility performance of ECCs was achieved. Finally, using wollastonite with a 44:1 aspect ratio showed better performance characteristics than wollastonite with a 30:1 aspect ratio. These findings show that a higher aspect ratio of synthetic wollastonite improves results. Therefore, synthetic wollastonite can be used because it has a higher aspect ratio than natural wollastonite. Although ECC shows superior performance characteristics compared to conventional concrete, it is costly due to the polyvinyl alcohol (PVA) fiber found in ECC and absence of coarse aggregate [33]. Wollastonite is a usable material for producing more economical ECC without losing the performance characteristics of ECC.

## 5. Geopolymer Concrete

**Figure 11:**
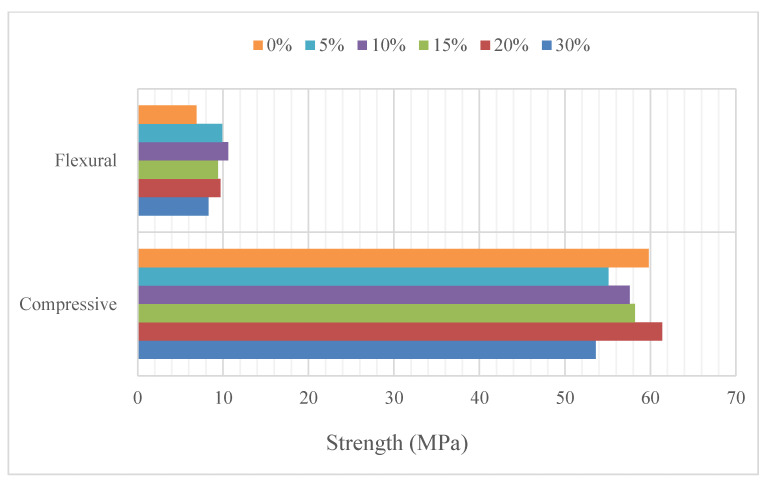
Compressive and flexural strengths of the mold-cast geopolymers at 7 days based on Ref. [49].

Geopolymer concrete is formed as a result of geopolymerization of aluminosilicate source materials (such as fly ash, furnace slag and metakaoline) with sodium- or potassium-sourced alkali activators [45,46]. Wollastonite was used as a precursor (such as fly ash slag or metakaolin), sand substitute or reinforcement with different material combinations in geopolymer concrete. When wollastonite was used instead of a precursor [47], usage of 10% wollastonite increased the strength properties in the range of 18–20%. In another study, in which wollastonite was used instead of both sand and a precursor [48], workability and setting time decreased with wollastonite. When wollastonite was used instead of sand, use of 10% wollastonite increased the compressive strength by 17%, and use of 20% wollastonite increased the flexural strength by 43% compared to the control mixture. While usage of wollastonite instead of a precursor did not enhance the flexural strength, use of 10% wollastonite increased the compressive strength by approximately 17% compared to the control mixture. Bong et al. [49] used wollastonite instead of only sand and found that the setting time was reduced. Figure 11 shows the compressive and flexural strengths of the mold-cast geopolymers at 7 days, and a 10% replacement level was found as the optimum ratio, and, at this rate, the flexural strength increased by 54%, but the compressive decreased by 4% compared to the control mixture. The wollastonite was partially dissolved in the mixture because of an alkaline environment. Acicular wollastonite was partially bonded to the geopolymer matrix, strengthened the matrix and enhanced the flexural strength.

In a study where wollastonite was used as a reinforcement, different material combinations were also tested in addition to wollastonite [50]. Substitution of metakaolin with 5% wollastonite, 5% tremolite and 2% of short basalt fiber reinforcement increased the compressive strength. In a study where polypropylene fiber, polyvinyl alcohol fiber and wollastonite were tested, usage of polypropylene fiber, polyvinyl alcohol fiber and wollastonite instead of metakaolin was examined: 1% polypropylene fiber + 1% polyvinyl alcohol fiber and 15% wollastonite + 2% polyvinyl alcohol fiber showed the highest compressive strengths [51]. The rates of increase were 90% and 160%, respectively. It also increased flexural strength and sulfate attack resistance. In a study where wollastonite and glass fiber were used instead of metakaolin, use of wollastonite enhanced its viscosity and mechanical properties [52]. Vishnu et al. [53] used wollastonite and graphene oxide in a self-compacting geopolymer. It was found that wollastonite performed better with graphene. The improvement in the microstructure of the geopolymer matrix through mechanical interlocking of unreacted wollastonite particles and bonding to the geopolymeric gel was attributed to the improvement in the geopolymer’s performance properties [48]. Geopolymer has attracted much attention in recent years as an alternative construction material to concrete. The use of wollastonite in geopolymer can be an applicable solution to overcome the disadvantage of lower strength than concrete and improve the performance features.

## 6. Self-Compacting Concrete

**Figure 12:**
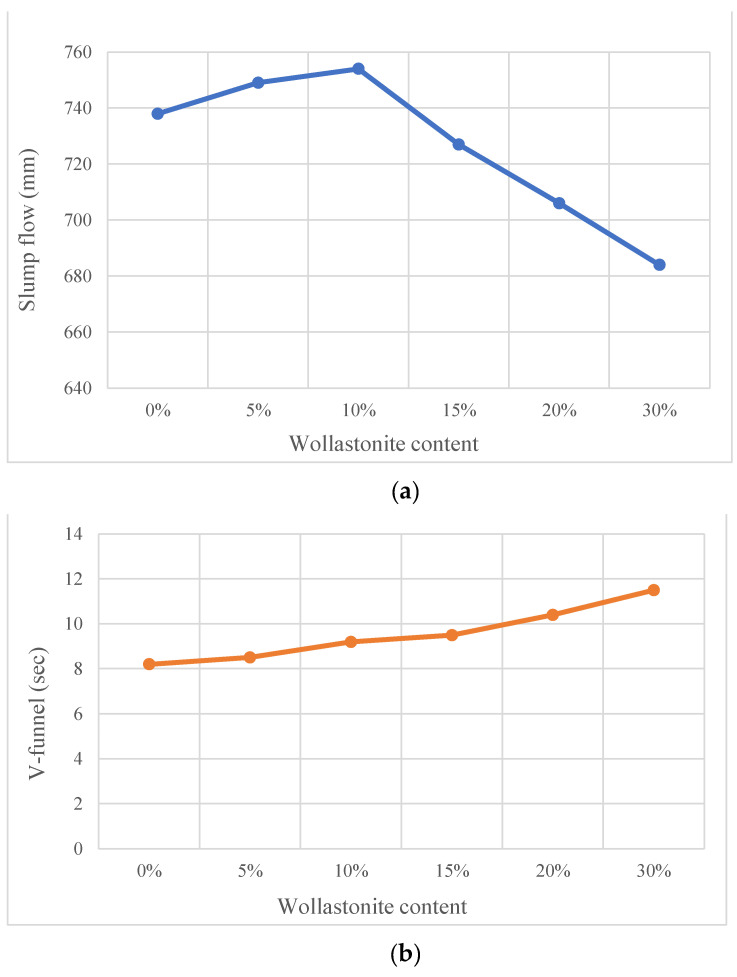
(**a**) Slump flow and (**b**) v-funnel of results of wollastonite based on Ref. [55].

Self-compacting concrete is a concrete that can be compacted without requiring any labor and fills the formwork homogeneously thanks to its self-flowing feature [54]. Jindal et al. [54] used wollastonite instead of sand and found that cohesiveness increased and water absorption decreased. The use of wollastonite at rates of up to 30% increased the flexural strength, while it did not have a positive effect on compressive strength, but it is comparable to the control mixture. In the study where wollastonite was used instead of cement, a control mixture was formed by using slag instead of 20% cement. In addition to usage of slag, wollastonite was used from 5% to 25% by 5% steps [55]. The workability results of this study are provided in Figure 12. Workability decreased with usage of wollastonite. The acicular structure of wollastonite and the particle size of wollastonite smaller than the particle size of cement are the reasons for decreasing workability [13]. Usage of 15% wollastonite increased compressive and flexural strength. In addition, its durability properties enhanced with the usage of wollastonite. In the study where the combination of wollastonite, fly ash and microsilica was studied, the use of almost equal amounts of wollastonite, fly ash and microsilica instead of about 30% cement had a positive effect on mechanical strength and durability properties [56]. Although it is advantageous to self-compacting concrete without requiring labor, a porous structure that could occur may cause durability problems. The filling effect of wollastonite has a positive effect on durability performance. It is observed that the durability properties of self-compacting concrete were also improved with utilization of wollastonite in the studies.

## 7. Ultra-High-Performance Concrete

**Figure 13:**
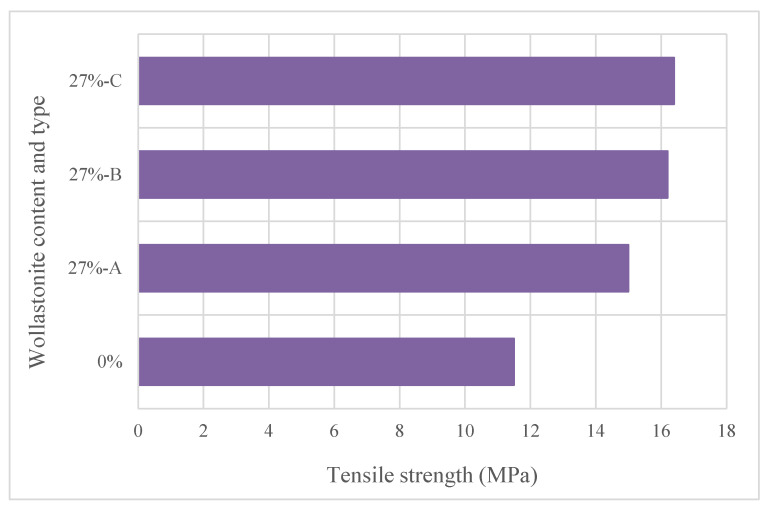
Tensile strength of 27% substitution of different types of wollastonite based on Ref. [57].

Ultra-high-performance concrete (UHPC) is defined as a type of concrete with very high strength and durability. Kwon et al. [57] used wollastonite, which has different aspect ratios, at 0%, 10%, 20% and 27% ratios instead of sand in UHPC. The tensile strength of 27% substitution of different types of wollastonite is shown in Figure 13. This increase could be due to bonding of the wollastonite microfibers and the mortar by a hydration reaction in which liberated Ca(OH)_2_ reacts with SiO_2_ in the wollastonite microfibers and forms C–S–H [20,57]. The mechanical properties (tensile strength, strain capacity and energy absorption capacity) improved with the use of wollastonite. The authors also stated that use of a low aspect ratio should be avoided. A high aspect ratio was also emphasized in the study conducted for ECC [33]. These findings show that a high aspect ratio performs better in cementitious composites. In a study where wollastonite was used instead of cement, wollastonite was tried instead of cement at a rate of 4%, 8% and 12% [21]. An increase was observed in compressive strength, hydration process and cracking resistance; a decrease was observed in lower shrinkage strains, but no significant improvement was observed in flexural strength. Soliman and Mehdi [58] examined the effect of wollastonite as a cement substitute as well as the effect of shrinkage-reducing admixture. Wollastonite increased the compressive strength and also mitigated the compressive strength decrease caused by the use of shrinkage-reducing admixture. Zareei et al. [59] formed two mixture groups: only using wollastonite instead of cement and also usage of wollastonite instead of cement+recycled waste ceramic aggregate instead of coarse aggregate. Workability, water absorption and compressive strength decreased with the use of wollastonite. Usage of wollastonite enhanced the splitting tensile strength, flexural strength and modulus of elasticity of concrete. The use of minimum cement or aggregate with utilization of wollastonite is valuable to obtain sustainable UHPC without losing the performance characteristics of UHPC.

## 8. Pavement Concrete

**Figure 14:**
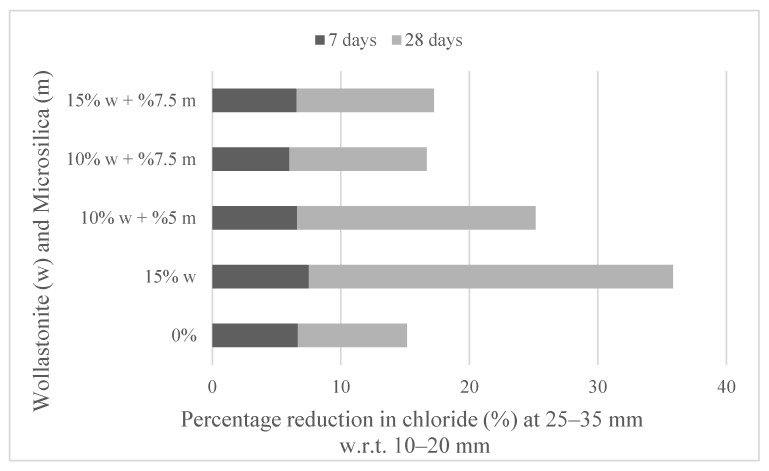
Percentage reduction in chloride and wollastonite–microsilica replacement based on Ref. [27].

Pavement is the top layer of the superstructure and forms a smooth rolling surface for vehicles. Due to the positive effect of wollastonite on the performance properties of concrete, studies were carried out on its use in pavement. Ransinchung and Kumar [60] used wollastonite instead of cement in their study. The use of 10% wollastonite increased by 11% and the use of 20% wollastonite increased by 5.2% on the 28-day compressive strength compared to the control mixture. The use of 10% wollastonite increased the 28-day flexural strength by 20.5% compared to the control mixture. The use of 20% and 30% for flexural strength also provided better results than the control mixture. The increase in flexural strength was higher than compressive strength. In another study, wollastonite was used instead of sand, and, in addition, fly ash was used instead of cement [61]. It was found that wollastonite reduces the slump value of fresh concrete and increases its density, abrasion resistance and compressive and flexural strength. It was emphasized that the combination of wollastonite + fly ash is also a usable alternative. The findings of these studies suggest that wollastonite could be a suitable material to be used in rigid pavements [60,61] and that the combination of wollastonite + fly ash can be used in both conventional concrete [39,43] and pavement [61], and, finally, it was indicated that it could be a suitable alternative material combination. Ransinchung et al. [27] examined the effect of wollastonite, microsilica and a combination of wollastonite + microsilica for pavement, similar to the study conducted for mortar [20]. The percentage reduction in chloride and wollastonite–microsilica replacement is shown in Figure 14. As a result of the saturated water absorption, rate of water absorption, coefficient of water absorption and chloride ion penetration durability experiments, it was stated that up to 15% wollastonite and 7.5% microsilica enhance its durability properties. The use of wollastonite in concrete pavements provides advantages in terms of cost, strength and durability. The use of wollastonite in pavement concrete is crucial in order to reduce its high cost compared to flexible pavement and to make its use widespread. 

Wollastonite uses and different effects are summarized in Table 3. Some studies are provided to show the effect of using wollastonite on different properties. It is observed that both natural and synthetic wollastonite enhance the mechanical and durability properties of cement-based composites. It has been determined that wollastonite is a material that can be used instead of both aggregate and cement for sustainable concrete production. 

## 9. Conclusions

Decreasing the usage of cement and aggregate is a crucial and currently relevant challenge. As an alternative to cement and aggregate, pozzolanic materials have been used for many years and have positive effects on cement-based composites. Not only pozzolanic materials but also inert materials can be used instead of cement and aggregate. One of the inert materials used in cement-based composites is wollastonite, which has two main basic components (CaO and SiO_2_). Wollastonite (calcium metasilicate-CaSiO_3_) has been Investigated by many researchers for approximately the last two decades. Moreover, artificially synthesized wollastonite is a novel and high-quality inert material because of the higher aspect ratio and the possibility of production by using different materials. This study systematically presented the use of natural and synthetic wollastonite in cement-based composites. The use and effects of wollastonite in cement-based composites are as follows:

- In the use of cement paste and mortar of wollastonite, rates of 3–10% usage instead of cement demonstrated a positive effect, while this rate increased up to 30% in sand. Wollastonite and different material combinations also provided applicable results. The use of synthetic wollastonite instead of cement by up to 10% for improving the performance characteristics of mortar is crucial for sustainability.
- In the range of 10–15%, use of wollastonite instead of cement enhanced the mechanical and durability properties of conventional concrete. The positive effect of using a combination of fly ash and wollastonite up to 60% was also available in the studies.
- Superior performance characteristics of engineered cementitious composite (ECC) are further enhanced with wollastonite, and 6% wollastonite substitution was stated as the optimum ratio. In addition, synthetic wollastonite with high aspect ratios of 44:1 and 33:1 was tested. The synthetic wollastonite with an aspect ratio of 44:1 showed better performance than 33:1. The effect of high aspect ratio was observed on ECC.
- Wollastonite was used instead of a precursor and sand in geopolymer concrete. The low mechanical property disadvantage of geopolymer was partially eliminated with the utilization of wollastonite. Strength increases were achieved with the use of 10–20% instead of sand.
- Positive performance properties were obtained from self-compacting concrete produced with wollastonite, both alone and in combination with different materials, up to 30% replacement.
- In ultra-high-performance concrete, the usage of wollastonite 27% instead of sand and up to 12% instead of cement increased the performance properties. Wollastonite can also increase sustainability without losing its performance properties.
- Only wollastonite and different material combinations were tested in pavements. Positive effects were observed in the use of up to 15% wollastonite.

It has been proven by studies that wollastonite is a material with an inert structure, and, therefore, it behaves similar to a fiber in cementitious composites due to its acicular particle structure. The use of wollastonite in cement-based composites both improves mechanical-durability properties and reduces the use of cement and aggregate, therefore providing more sustainable production. Especially regarding synthetic wollastonite’s high aspect ratio and different material usage possibilities, its use up to 10% instead of cement is promising. In future studies, some durability tests (freeze–thaw, corrosion, etc.) of using wollastonite can be performed for all cementitious composites. The effect of synthetic wollastonite on different cement-based composites (except mortar and ECC) and use of synthetic wollastonite instead of sand can be studied. Moreover, the life cycle assessment of cementitious composites produced with synthetic wollastonite can be investigated. Finally, synthetic wollastonite and different pozzolanic material combinations can be tested.

## Figures and Tables

**Table 1:** Chemical composition of wollastonite based on Ref. [7].

Chemical Analysis (%)	Wollastonite
CaO	44.55
SiO_2_	50.78
Al_2_O_3_	0.83
Fe_2_O_3_	0.17
MgO	0.47
SO_3_	0.04
K_2_O	0.001
Na_2_O	0.363
TiO_2_	0.49

**Table 2:** Mix design of concrete (kg/m^3^) based on Ref. [44].

Mix	W/b Ratio	OPC	* WF	CA	FA	* WGF	Water	* SPDosage %
1	0.35	425	0	1298	628	0	148.75	1.25
2	0.35	382.5	42.5	1298	565.2	62.8	148.75	1.3
3	0.35	382.5	42.5	1298	502.4	125.6	148.75	1.5
4	0.35	382.5	42.5	1298	439.6	188.4	148.75	1.65
5	0.35	382.5	42.5	1298	376.8	251.2	148.75	1.9
6	0.35	382.5	42.5	1298	314	314	148.75	2.1

* WF = wollastonite fiber, WGF = waste granite fines, SP = high water reducing superplasticizer.

**Table 3:** Usage and different effects of wollastonite in the literature.

Study in Literature	Wollastonite Replacement	Effect of Wollastonite
[34]	5–10% instead of cement	Increment about 34% fracture toughness
[35]	0–15% instead of cement	Increment about 12% compressive and increment about 6% flexural strength
[36]	0–50% instead of cement	Decrease in workability and increment in cement hydration
[28]	5–15% instead of cement	Increment in crack growth resistance and ductility
[37]	10–30% instead of cement and sand	Increment in initial setting time and decrease about 47% drying shrinkage
[7]	0–12% instead of cement (synthetic wollastonite)	Increment about 8% compressive and increment about 11% flexural strength; decrease about 15% water sorptivity coefficient, about 4% rapid chloride permeability and about 25% gas permeability
[32]	0–25% instead of cement	Decrease in porosity, water permeability, chloride diffusion and carbonation depth
[43]	0–25% instead of cement	Increment in resistance against corrosion

## Data Availability

Not applicable.
